# Intracholecystic Papillary Neoplasm: A Case Report

**DOI:** 10.7759/cureus.66309

**Published:** 2024-08-06

**Authors:** Sham Taqwa, Daiki Soma, Kathleen Ehresmann, Ashraf El-Hinnawi

**Affiliations:** 1 Surgery, University of Jordan, Amman, JOR; 2 Transplant and Hepatobiliary Surgery, University of Florida College of Medicine, Gainesville, USA

**Keywords:** hepatobiliary interventions, cancer gallbladder, robotic assisted cholecystectomy, transplant and hepatobiliary surgeon, gallbladder polyp

## Abstract

We present a case of a 30-year-old female with symptomatic gallstones and associated gallbladder polyps. An incidental finding of intracholecystic papillary neoplasm (ICPN) with high-grade dysplasia was found after pathological examination of the gallbladder after robotic cholecystectomy. This rare condition can be associated with malignant transformation. In this case report, we discuss this rare entity and share our experience and review of the literature.

## Introduction

Intracholecystic papillary neoplasm (ICPN) is a rare condition. We report a case of gallbladder polyp requiring cholecystectomy, with a postoperative pathology showing ICPN. We discuss the diagnosis, types, management, and follow-up of this rare condition, as well as a review of the literature [1-3].

## Case presentation

**Figure 1:**
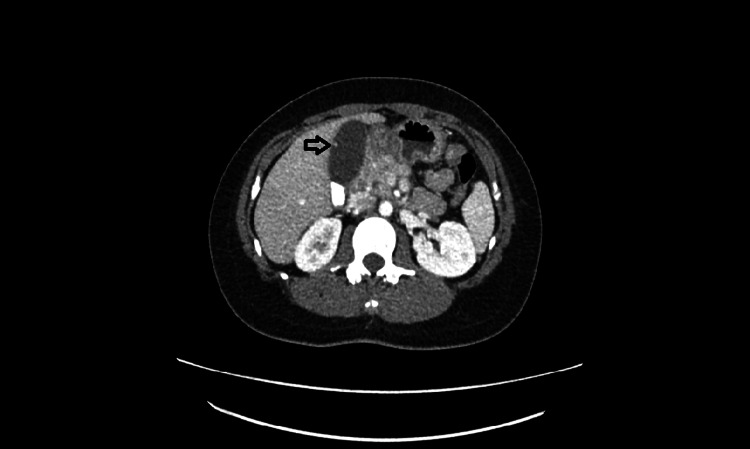
Small gallbladder polyp seen on computed tomography.

A 30-year-old woman presented to her primary care provider with complaints of one day of right upper quadrant abdominal pain, bloating, nausea, and decreased appetite. A right upper quadrant ultrasound was obtained, which demonstrated cholelithiasis without cholecystitis and an 8-mm broad-based echogenic focus favored to represent a gallbladder polyp. A sonographic Murphy’s sign was not elicited. The common bile duct was mildly dilated at 7 mm. A computed tomographic scan of the abdomen and pelvis showed cholelithiasis without acute cholecystitis and redemonstrated the small gallbladder polyp seen on ultrasound (Figure 1). She was referred for a surgical evaluation, and a cholecystectomy was recommended.

**Figure 2:**
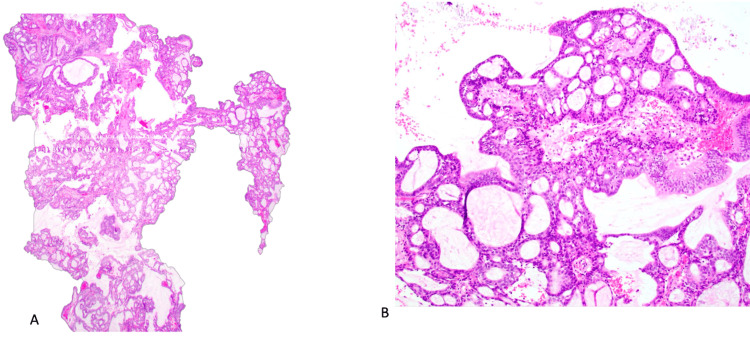
Intracholecystic papillary neoplasm. (A) Low magnification shows epithelial proliferation in papillary, tubular, and tubulopapillary patterns with limited stroma. (B) Higher magnification shows a biliary phenotype with cuboidal cells showing eosinophilic cytoplasm, enlarged nuclei, and prominent nucleoli consistent with high-grade dysplasia.

The patient underwent an uncomplicated robotic cholecystectomy and was discharged home on the day of surgery. Surgical pathology confirmed chronic cholecystitis, and the gallbladder specimen contained a 1.0 cm IPCN of the gallbladder and a pyloric gland adenoma (PGA) with high-grade dysplasia and a negative cystic duct margin (Figure 2).

Her case was presented at our institution’s multidisciplinary GI tumor board conference for a review of radiology and pathology. The consensus recommendation indicated that no additional treatment was necessary as the pathology revealed no cancer. She was seen in the clinic for a three-month postoperative visit with MRCP, at which time she was doing well clinically and had returned to her preoperative baseline function. The MRCP showed no hepatic lesions or metastatic disease in the abdomen.

## Discussion

This case is a rare presentation of a 30-year-old female incidentally diagnosed with ICPN of the gallbladder, PGA, and high-grade dysplasia following cholecystectomy for symptomatic cholelithiasis. The clinical significance of these concurrent findings in a patient of this age group warrants careful consideration.

PGA of the gallbladder is a polypoid, preinvasive epithelial neoplasm characterized by uniform back-to-back pyloric glands organized in a tubular configuration. ICPN, another grossly visible preinvasive neoplasm of the gallbladder, can be subclassified into four morphological subtypes: biliary, gastric, intestinal, and oncocytic. ICPN is more frequently diagnosed in women over 60 years old and is seen in only 0.4% of cholecystectomies [1]. These uncommon tumors typically manifest as intramucosal papillary or polypoid masses, often accompanied by mucin overproduction.

The varying degrees of dysplasia observed in ICPN demonstrate the adenoma-carcinoma sequence, differing from the presumed de novo carcinogenesis pathway seen in papillary adenocarcinoma. Consequently, from a carcinogenic standpoint, ICPN is distinct from papillary adenocarcinoma, with each tumor type displaying unique prognostic characteristics. Notably, ICPN rarely infiltrates or metastasizes [2,3].

Although 50% of patients with ICPN exhibit invasive malignant components, the overall prognosis remains favorable. Patients with non-invasive ICPN demonstrate promising survival rates at one, three, and five years, with rates of 90%, 90%, and 78%, respectively [1]. Furthermore, even individuals with associated invasive carcinoma exhibit significantly better clinical outcomes compared to those diagnosed solely with conventional invasive gallbladder cancer.

The only known effective therapy for adenomas of the gallbladder is cholecystectomy. This should be considered when the adenoma is >10 mm on ultrasound, in cases of multiple lesions, in patients over 50 years of age, and in association with gallstones, as in our patient’s case.

The coexistence of our patient’s intracholecystic papillary neoplasm, pyloric gland adenoma of the gallbladder, high-grade dysplasia, and cholelithiasis raises intriguing questions regarding their interplay. Literature on such cases is scarce, underscoring the need for a comprehensive understanding of this rare combination of gallbladder pathologies. Additionally, our patient's medical history, which includes a previous episode of streptococcal pharyngitis with abscesses, adds complexity, although a direct link between these past infections and the current gallbladder findings is not apparent.

Examination of existing literature reveals few precedent cases with similar combinations of intracholecystic papillary neoplasm, pyloric gland adenoma of the gallbladder, and high-grade dysplasia, particularly in younger patients. This case is significant not only because of its rarity but also because of its potential implications for diagnostic and therapeutic approaches.

Clinical management considerations must address the unique challenges posed by multiple pathologies. The incidental discovery of intracholecystic papillary neoplasm during cholecystectomy underscores the importance of a thorough pathological examination of gallbladder specimens. We suspect genetic predisposition, environmental factors, and a complex interplay of pathogenic mechanisms can explain the observed coexistence of this patient’s cholelithiasis, intracholecystic papillary neoplasm, pyloric gland adenoma of the gallbladder, and high-grade dysplasia. However, definitive conclusions about causality remain elusive.

The limitations of this study include its single-case nature and the absence of a larger cohort for comparison. Generalizability is limited, and caution should be exercised when extrapolating these findings to broader populations.

## Conclusions

ICPN is a rare condition. We present an incidental finding of ICPN in a gallbladder polyp. Knowledge about the condition and having a reasonable level of suspicion can result in the early detection of this rare condition. Early detection can potentially prevent advanced disease and can be associated with better patient care and outcomes.

## References

[1] Adsay V, Jang KT, Roa JC (2012). Intracholecystic papillary-tubular neoplasms (ICPN) of the gallbladder (neoplastic polyps, adenomas, and papillary neoplasms that are ≥1.0 cm): clinicopathologic and immunohistochemical analysis of 123 cases. Am J Surg Pathol.

[2] Natov NS, Horton LC, Hegde SR (2017). Successful endoscopic treatment of an intraductal papillary neoplasm of the bile duct. World J Gastrointest Endosc.

[3] Muranushi R, Saito H, Matsumoto A (2018). A case report of intracholecystic papillary neoplasm of the gallbladder resembling a submucosal tumor. Surg Case Rep.

